# Nature’s Methods

**Published:** 1881-10

**Authors:** 


HALL'S
### JOURNAL OF HEALTH
AND
MISCELLANY.

E. H. GIBBS, A. M., M. D. - - EDITOR.

Founded in 1854, and published Monthly without Interruption since that Time.

### NATURE'S METHODS.

MANY diseases, when they once fasten on the system, will run a regular course in spite of the best efforts of the most skillful physician. It must not be supposed, however, that he can be dispensed with. Medical men have done more than any other class to prolong the average term of human life; not so much by direct interference with the course of disease, as by modifying and controlling it. The office of the physician is to remove obstructions and leave nature to repair the ravages made by disease or accident, and this is all the wise physician will claim to be able to do; for with all his knowledge, he knows very little of nature's methods. The action of all medicines is, to a great extent, empirical, but it is only the ignorant and inexperienced that are "Empirics." The true physician knows from experience and from study that certain remedies will, as a rule, produce certain effects, and he also knows that through errors in diagnosis, that the wisest are liable to make, he is often disappointed as to results.

It is difficult to make comparisons between two plans of action whose harmony is often essential to the saving of life; but were an invalid compelled to choose between good nursing, without skillful medical aid, or skilled medical aid without good nursing, it would be far safer to choose the former and trust the case to nature—the only physician that never errs. And just here it occurs to me to make a suggestion that it would be well to remember.

I was often called to cases that taxed my ingenuity before experience taught me how to manage them satisfactorily. A good soldier when summoned to battle expects to find an enemy to fight, but a good doctor may not be able to find the enemy. The most careful investigation sometimes fails to discover any disease, and all the vital organs seem to be working harmoniously; yet the patient is languid and nervous, with pains in the head, back and limbs. The family and friends are anxious that something should be done; but strictly speaking, there is nothing for the physician to do. There are cases where a little deception might be justifiable. Some patients are never satisfied unless they have something bitter to swallow at stated periods. So long as it is harmless—a mere placebo—it may be an easy way to accomplish a desirable object, to satisfy the mind, but the best thing possible is to have the nurse or some member of the family bathe the head, back and limbs with bay rum, or dilute alcohol, rubbing thoroughly with the hands. This treatment is always grateful, and often completes the cure. Nature is thus aided in her efforts to promote a healthy circulation of the blood, and it is by such gentle means that the aid becomes effective. It is often necessary to carry this plan a little further and to supplement the rubbing with plasters of mustard to the back of the neck or between the shoulders, moving them about every few minutes when smarting too severely.

Warm foot-baths are often useful and seldom come amiss. The headache and uneasiness often depend upon an empty stomach, even when the patient becomes nauseated at the thought of food; or the nausea may depend upon the remembrance of some miserably prepared soup or broth that had at some time disgusted him. I have no doubt but such apologies for nourishing foods have frequently been the cause of serious relapses. The utmost care should be observed in the preparation of food for invalids, as well as the most scrupulous neatness. The invalid's eyes are sharp and his senses acute and easily disturbed. Every dish must be seasoned to the taste and made most palatable. Knives, forks and spoons should be bright and spotless and the dishes clean. I have seen a patient who was suffering for food powerless to swallow a spoonful of soup, because a little had been allowed to get on the edge of the plate. It is to be regretted that so little attention is usually paid to these things, that are of so little consequence to those who are in health, but are of vital importance to the sick.

---

Experiments in growing tea in this country show that while we can't raise much tea, we can produce an herb that for poisonous and deadly effect cannot be rivalled.—*Boston Post.*

Eli Perkins was called a liar by a South Bend man, and Eli promptly challenged him to a duel. When the fatal hour arrived, Eli was in Chicago and the other man was in Indianapolis.—*Detroit Free Press.*

